# Estimation of PM_2.5_ Concentration Efficiency and Potential Public Mortality Reduction in Urban China

**DOI:** 10.3390/ijerph15030529

**Published:** 2018-03-15

**Authors:** Anyu Yu, Guangshe Jia, Jianxin You, Puwei Zhang

**Affiliations:** School of Economics and Management, Tongji University, Siping Road 1500, Shanghai 200092, China; yuanyu1990yy@163.com (A.Y.); jiagsh803@tongji.edu.cn (G.J.); yjx2256@vip.sina.com (J.Y.)

**Keywords:** slacks-based measure, data envelopment analysis, PM_2.5_ concentration, public mortality, integrated exposure risk, potential reduction

## Abstract

The particulate matter 2.5 (PM_2.5_) is a serious air-pollutant emission in China, which has caused serious risks to public health. To reduce the pollution and corresponding public mortality, this paper proposes a method by incorporating slacks-based data envelopment analysis (DEA) and an integrated exposure risk (IER) model. By identifying the relationship between the PM_2.5_ concentration and mortality, the potential PM_2.5_ concentration efficiency and mortality reduction were measured. The proposed method has been applied to China’s 243 cities in 2015. Some implications are achieved. (1) There are urban disparities in estimated results around China. The geographic distribution of urban mortality reduction is consistent with that of the PM_2.5_ concentration efficiency, but some inconsistency also exists. (2) The pollution reduction and public health improvement should be addressed among China’s cities, especially for those in northern coastal, eastern coastal, and middle Yellow River areas. The reduction experience of PM_2.5_ concentration in cities of the southern coastal area could be advocated in China. (3) Environmental consideration should be part of the production adjustment of urban central China. The updating of technology is suggested for specific cities and should be considered by the policymaker.

## 1. Introduction

The dramatic processes of economic growth and urbanization have resulted in serious air pollution issues in China. It is reported that less than 1% of the 500 largest cities in China have achieved the recommended air quality standards and seven out of the ten most polluted cities in the world are in China [1]. A significant reflection of the emerging air pollution is the increased foggy and hazy days presently occurring [2]. It has been stated that the number of annual foggy and hazy days in China has increased from four to 18 during 1961–2012 [3]. The increasingly foggy and hazy weather does not only disturb normal traffic, but also damages the health of residents [4]. Several studies have detected a causal relationship between air pollution and public health risk [5,6,7]. For example, it has been observed that seven million people died from the combined effects of household and ambient air pollution worldwide in 2012 [8]. 

Particulate matter 2.5 (i.e., PM_2.5_) is defined as the mass concentration of particles with aerodynamic diameters smaller than 2.5 microns [2], and its increasing trend could be regarded as one important cause of increasing annual foggy and hazy days in China [9]. Eighty-three percent of the population in 2012 lived in the regions where the annual mean PM_2.5_ concentration exceeded the ambient air quality standard in China [10]. China’s excessive PM_2.5_ concentration is mainly derived from the combustion of fossil fuels, especially coal [4]. Due to the massive coal-dominant energy consumption structure [4], China’s energy consumption (i.e., 2.43 billion tons of oil equivalent) has surpassed the U.S. (i.e., 2.29 tons of oil equivalent) [11] to be the world’s largest since 2010. China’s huge coal consumption has worsened the PM_2.5_ pollution performance, both from stationary sources (power plant, chemical plant, etc.) and moving sources (motor vehicle, off-road machines, etc.) [12]. To mitigate China’s foggy and hazy days, reduction of PM_2.5_ pollution needs to be addressed. China’s 13th five-year plan states that cities at prefecture level and above should reduce heavy air pollution days by 25% and strengthen PM_2.5_ pollution control. Meanwhile, emission-reduction solutions have been proposed in the academic field, for example, advocating the usage of renewable energies and public transportation [13], regulating behaviors to reduce household PM_2.5_ emission [14], enhancing technology progression, and updating industrial infrastructure [15]. 

To launch reasonable PM_2.5_ mitigation solutions, the performance of PM_2.5_ pollution needs to be measured. Generally, the PM_2.5_ concentration is regarded as a proxy of the pollution performance [16]. However, this study believes that reasonable PM_2.5_ pollution performance evaluation for emission reduction should not only consider the physical concentration, but also all the influential production factors, such as the population density and economic output. It is not reasonable to stop production for PM_2.5_ concentration reduction in underdeveloped regions, for example, since the PM_2.5_ emission caused by energy consumption indirectly results in economic growth. Conflicts may exist between the PM_2.5_ concentration reduction and economic growth. Furthermore, there are geographic disparities in China due to its vast territory and diverse economic levels. Mitigation of PM_2.5_ concentration could be affected by regional diversity, as are economic output and population density. Thus, a regional diversity analysis of total-factor performance should be undertaken. 

Data envelopment analysis (i.e., DEA) is a “data-oriented” approach for evaluating the performance of a set of peer entities, which converts multiple inputs into multiple outputs [17]. It is a widely accepted total-factor evaluation because it forbids presetting of production functions to avoid the misspecification of risks [18]. It is particularly popular in environmental applications. The environmental efficiency estimated by DEA is a widely accepted environmental performance measure (e.g., [19,20,21]), which could be defined as the ability to create more goods and services while using fewer resources and creating less negative environmental impact [22]. There are several studies measuring the environmental efficiency at the macro level. The literature can be divided into two streams. The first evaluates the environmental efficiency at the regional scale, considering countries or states [23,24,25]. The second assesses the environmental efficiency of industrial sectors, especially for pollution intensive industries [26,27,28]. Moreover, issues such as PM_2.5_ emission allocation [29], influential factor analysis [30], input optimization for emission reduction [12], and emission abatement cost calculation [31] based on environmental efficiency have been previously proposed.

Based on environmental efficiency estimation, the efficiency representing the PM_2.5_ concentration performance (i.e., PM_2.5_ concentration efficiency) can be identified by DEA in this study. PM_2.5_ concentration efficiency, as a special kind of environmental efficiency, is defined in this study as the PM_2.5_ contribution to the economic and sustainable development of the macro-regional production system. This is an effective way to measure the expected PM_2.5_ concentration performance by producing more economic output while using fewer resources. To the best of the authors’ knowledge, there are limited studies regarding environmental efficiency estimation related to PM_2.5_ concentration [12,29,32,33]. The existing studies mainly focus on environmental efficiency but not PM_2.5_ concentration efficiency. The only exception is Wu et al. [29], who estimated the allocative efficiency in PM_2.5_ emission and concentration in China during 2001–2010. Unfortunately, that study measures PM_2.5_ efficiency at the provincial level. The PM_2.5_ concentration data is collected from urban monitoring sites and the reduction solutions are adopted based on the urban condition. A provincial evaluation cannot provide a detailed solution for cities within the province, which still leaves the research gap for an investigation at the city level. 

Furthermore, another important issue that exists in the study of PM_2.5_ concentration is public mortality derived from PM_2.5_ pollution. PM_2.5_ emissions are of small size and large surface area, so many viruses and bacteria can adhere and thus enter the lungs through the respiratory system without being blocked by the nasal cavity and the throat [34,35,36]. Several diseases such as ischemic heart disease, cerebrovascular disease, and chronic obstructive pulmonary disease have been proven to be caused by PM_2.5_ emission [37,38]. This paper aims to estimate potential PM_2.5_ concentration reduction and the corresponding reduction in public mortality. 

As its main contributions, this paper proposes a slacks-based DEA model to evaluate the PM_2.5_ concentration efficiency among all possible Chinese cities. The slacks-based model is first extended by considering the targets from both overall and environmental perspectives. The potential reductions of PM_2.5_ concentration in both perspectives are further discussed. Moreover, an integrated exposure risk (IER) model is adapted to identify the relationship between the PM_2.5_ concentration and public mortality. For the first time, the potential public mortality reduction by reduction of PM_2.5_ concentration is investigated by DEA. The proposed models are applied to an empirical study including 243 Chinese cities in 2015. Some policy suggestions for PM_2.5_ concentration reduction and public health improvement are proposed. 

The paper is structured as follows. Section 2 presents the variables, data sources, and method framework (i.e., the DEA and IER models). Section 3 conducts the empirical analysis to analyze the efficiency and potential reductions in urban China. The discussion and conclusion are further provided in Section 4 and Section 5.

## 2. Data and Methods

### 2.1. Observed Regions and Data Source 

**Figure 1:**
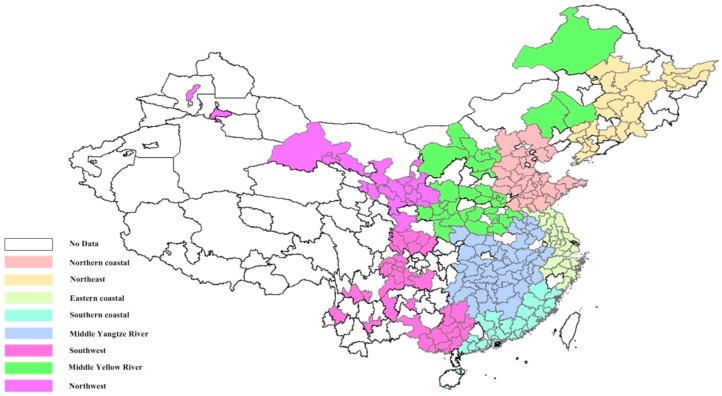
The illustration of the selected urban observations divided by areas.

The cities in mainland China can be classified into different areas according to the regional diversity in economy and politics [39]. There are 31 provinces, autonomous regions, and municipalities in mainland China, which can be aggregated into eight areas based on their geographic locations [40]. An illustration is shown in Figure 1. Notably, there are recorded annual data of PM_2.5_ concentration collected from ground monitoring sites among prefecture-level and above cities since 2013. Seventy-four and 161 cities in China are observed to have recorded PM_2.5_ concentrations in 2013 and 2014, respectively. While 338 cities have recorded PM_2.5_ concentrations in 2015, only 243 have available data regarding all the production indicators. Therefore, 243 cities in 29 regions (urban data is not available in Tibet and Chongqing) in 2015 are selected as observations. The PM_2.5_ concentration data from 2015 is used in this study.

In this study, the selected indicators are labor, capital, energy consumption, Gross Domestic Production (GDP), and PM_2.5_ concentration. The data of GDP, labor, and energy consumption for urban districts were collected from the China City Statistical Yearbook in 2016. Following Wang et al. [41], the energy consumption data in this study consist of the consumption of natural gas, liquefied petroleum gas, and electric power. Since capital stock data is not available in any existing database, this study adopts the perpetual inventory method to estimate the urban capital stock at the 2002 constant price by following Liu et al. [42] and Xu [43]. The monthly PM_2.5_ concentration is collected from the “online monitoring and analysis platform for the air quality in China” [44], which is linked to the database of the China national environmental monitoring center. The annual PM_2.5_ concentration is obtained by the proposed method in Hao et al. [45]. The descriptive statistics of the obtained data from 2015 is summarized in Table 1.

### 2.2. Methods

A slacks-based model for evaluating the PM_2.5_ concentration efficiencies of regional production systems is proposed in this section. The PM_2.5_ concentration efficiencies are optimized by two target functions from both overall and environmental perspectives, which can help to identify the environmental efforts in regional production. The proposed potential reduction in PM_2.5_ concentration is based on the estimated efficiencies. Additionally, the potential public mortality reductions are measured by identifying the relationship between PM_2.5_ concentration and public mortality.

#### 2.2.1. Particulate Matter 2.5 Concentration Efficiency Estimation

Assume that there exist *n* decision making units (DMUs) as regional production systems (*RS_j_*, *j* = 1, ..., *n*). In the regional production process, labor (*L*), capital (*K*), and energy consumption (*E*) are non-energy inputs and energy input, respectively. Gross Domestic Production (*G*) is treated as the desirable output, and the undesirable output is PM_2.5_ concentration (*P*). Variable selection is taken from Sueyoshi and Yuan [32], but with some modifications. 

There are two types of models in DEA: radial and non-radial. Radial models deal with proportional changes of inputs or outputs. The non-radial slacks-based measure of efficiency (SBM) model discards the assumption of proportionate changes in inputs and outputs and deals with slacks directly [17]. The slacks-based measure (SBM) is widely adopted in environmental efficiency estimations by DEA [26,27] in the literature, because it can find the sources of inefficiency behind operational process by directly dealing with “input excess” and “output shortfall” [46]. The SBM model is aimed to evaluate the PM_2.5_ concentration efficiency as model (1).

(1) $$\begin{array}{ll} \theta_{i}= & \min(1-\frac{1}{3}(\frac{s_{l}^{-}}{L_{i}}+\frac{s_{k}^{-}}{K_{i}}+\frac{s_{e}^{-}}{E_{i}}))/(1+\frac{1}{2}(\frac{s_{g}^{+}}{G_{i}}+\frac{s_{p}^{-}}{P_{i}}))\\ s.t. & \sum_{j=1}^{n}\lambda_{j}L_{j}+s_{l}^{-}=L_{i},\\ & \sum_{j=1}^{n}\lambda_{j}K_{j}+s_{k}^{-}=K_{i},\\ & \sum_{j=1}^{n}\lambda_{j}E_{j}+s_{e}^{-}=E_{i},\\ & \sum_{j=1}^{n}\lambda_{j}G_{j}-s_{g}^{+}=G_{i},\\ & \sum_{j=1}^{n}\lambda_{j}P_{j}+s_{p}^{-}=P_{i},\\ & \sum_{j=1}^{n}\lambda_{j}=1,\\ & \lambda_{j},s_{l}^{-},s_{k}^{-},s_{e}^{-},s_{g}^{+},s_{p}^{-}\geq 0,\;j=1,2,\ldots ,n. \end{array}$$

(2) $$\begin{array}{ll} \theta_{i}= & \min(t-\frac{1}{3}(\frac{S_{l}^{-}}{L_{i}}+\frac{S_{k}^{-}}{K_{i}}+\frac{S_{e}^{-}}{E_{i}})\\ s.t. & t+\frac{1}{2}(\frac{S_{g}^{+}}{G_{i}}+\frac{S_{p}^{-}}{P_{i}})=1,\\ & \sum_{j=1}^{n}\eta_{j}L_{j}+S_{l}^{-}=tL_{i},\\ & \sum_{j=1}^{n}\eta_{j}K_{j}+S_{k}^{-}=tK_{i},\\ & \sum_{j=1}^{n}\eta_{j}E_{j}+S_{e}^{-}=tE_{i},\\ & \sum_{j=1}^{n}\eta_{j}G_{j}-S_{g}^{+}=tG_{i},\\ & \sum_{j=1}^{n}\eta_{j}P_{j}+S_{p}^{-}=tP_{i},\\ & \sum_{j=1}^{n}\eta_{j}=t,\\ & \eta_{j},S_{l}^{-},S_{k}^{-},S_{e}^{-},S_{g}^{+},S_{p}^{-}\geq 0,\;j=1,2,\ldots ,n. \end{array}$$

In model (1), $\theta_{i}$ is the evaluated overall efficiency for the evaluated region *i*, which is in the range of [0,1]. λ denotes the intensity variable for the participation of each Decision-Making Unit (DMU). $s_{l}^{-},s_{k}^{-},s_{e}^{-},s_{g}^{+},s_{p}^{-}$ are slack variables of labor, capital, energy, GDP, and PM_2.5_ concentration. The subscript “*i*” represents the evaluated regional production system. The constraint of $\sum_{j=1}^{n}\lambda_{j}=1$ indicates that the DEA model is in the variable return to scales form (i.e., VRS), which could be easily changed to be a constant return to scales form (i.e., CRS). The scale efficiency and returns to scale could be examined by the VRS form. Notably, the PM_2.5_ emission is mainly abated by application of technical instruments during the production process, which could be freely disposable in this study, as explained by Wang et al. [47]. Notably, model (1) is a non-linear form, which can be transformed into model (2) for simple calculation.

In model (2), the constraint is set as $\;t+\frac{1}{2}(\frac{S_{g}^{+}}{G_{i}}+\frac{S_{p}^{-}}{P_{i}})=1$. Then $s_{l}^{-},s_{k}^{-},s_{e}^{-},s_{g}^{+},s_{p}^{-}$ are transformed as $ts_{l}^{-}=S_{l}^{-},ts_{k}^{-}=S_{k}^{-},ts_{e}^{-}=S_{e}^{-},ts_{g}^{+}=S_{g}^{+},ts_{p}^{-}=S_{p}^{-}$. By solving model (2), optimal $t^{*},S_{l}^{-*},S_{k}^{-*},S_{e}^{-*},S_{g}^{+*},S_{p}^{-*}$ can be obtained to achieve the optimal overall efficiency $\theta_{i}^{*}$. If all the optimal slacks are equal to zero, the efficiency can be measured. The corresponding efficiency of PM_2.5_ concentration is measured as the ratio of expected PM_2.5_ concentration value to actual value [39], that is,

(3) $$\theta_{i}^{pm2.5}=(P_{i}-(S_{p}^{-}/t))/P_{i}$$

The $P_{i},\;S_{p}^{-}$ and t are optimal results obtained by solving model (2), and $\theta_{i}^{pm2.5}$ denotes the estimated PM_2.5_ concentration efficiency for region *i*.

#### 2.2.2. Particulate Matter 2.5 Concentration Efficiency Estimation from Overall and Environmental Perspectives 

Based on model (2) and Equation (3), PM_2.5_ concentration efficiency can be estimated, which is the overall efficiency of the specific regional production system. In other words, the PM_2.5_ concentration reduction in model (2) is treated as a partial target of the regional production. Thus, the PM_2.5_ concentration efficiency estimation of model (2) is from the overall perspective to achieve the optimal overall regional production performance. Another scenario is considered in this study, that is, estimation from an environmental perspective. From the environmental perspective, the only target of regional production is to obtain the optimal PM_2.5_ concentration efficiency. This means that regional production systems have made all the efforts to reduce PM_2.5_ concentration. Regarding the environmental perspective, model (1) could be transformed into the following model (4).

(4) $$\begin{array}{ll} \theta_{i}= & \max\frac{s_{p}^{-}}{P_{i}}\\ s.t. & \sum_{j=1}^{n}\lambda_{j}L_{j}+s_{l}^{-}=L_{i},\\ & \sum_{j=1}^{n}\lambda_{j}K_{j}+s_{k}^{-}=K_{i},\\ & \sum_{j=1}^{n}\lambda_{j}E_{j}+s_{e}^{-}=E_{i},\\ & \sum_{j=1}^{n}\lambda_{j}G_{j}-s_{g}^{+}=G_{i},\\ & \sum_{j=1}^{n}\lambda_{j}P_{j}+s_{p}^{-}=P_{i},\\ & \sum_{j=1}^{n}\lambda_{j}=1,\\ & \lambda_{j},s_{l}^{-},s_{k}^{-},s_{e}^{-},s_{g}^{+},s_{p}^{-}\geq 0,\;j=1,2,\ldots ,n. \end{array}$$

Model (4) is a modified form of the output-oriented SBM model with respect to the PM_2.5_ concentration [17]. The only difference between model (1) and model (4) is the target function. The DMUs launch all the efforts to reduce the PM_2.5_ concentration for the maximized target of $\left(s_{p}^{-}/P_{i}\right)$ in model (4). The PM_2.5_ concentration efficiency in model (4) (i.e., $(P_{i}-s_{p}^{-})/P_{i}$) is estimated from the environmental perspective, which is in the range of [0,1]). All the other elements in model (4) are the same as in model (1). 

Based on the obtained efficiency, the potential reduction of PM_2.5_ concentration is further estimated. Following Bian et al. [48], potential PM_2.5_ concentration reductions from the overall perspective (i.e., *PR_o_*) and environmental perspective (i.e., *PR_e_*) are calculated by Equations (5a) and (5b) based on models (3) and (4), respectively.

(5a) $$PR_{o}=S_{pi}^{o}/t,$$

(5b) $$PR_{e}=S_{pi}^{e}.$$

In which, $S_{pi}^{o}$ and $S_{pi}^{e}$ denote the slack variables of PM_2.5_ concentration in models (2) and (4), respectively. t means the transformed tool variable in models (2). Then the reduction gap between *PR_e_* and *PR_o_* (i.e., ∆*PR*) are determined as

(6) $$\Delta PR=PR_{e}-PR_{o}.$$

ΔPR reflects the potential reduction of PM_2.5_ concentration in ignorance of the economic target in the production, or, in other words, the additional reduction generated at the cost of economic output loss. It indicates that PM_2.5_ concentration reduction is treated as the most important consideration in the performance evaluation.

**Proposition** **1.** *The potential PM_2.5_ concentration reduction estimated from the environmental perspective is larger than or equal to that from the overall perspective.*

**Proof** **of** **Proposition** **1.** *It is clear that the optimal solution of slack variable*$\;S_{pi}^{o}\;$ *in model (2) is less than the* $S_{pi}^{e}$ *in model (4), since the target function in model (4) is as same as a partial target in model (1). With less limitation on the target function in model (4), the optimal solution* $S_{pi}^{e}$ *cannot be achieved by the model from the overall perspective. Thus, the potential reduction from the environmental perspective is larger than that from the overall perspective.*

#### 2.2.3. Potential Public Mortality Reductions 

Public mortality is negatively affected by PM_2.5_ concentration. The relationship between the PM_2.5_ concentration and mortality caused by diseases should be ascertained to determine the reasonable potential mortality reduction. The relationship can be explored by the IER model proposed in Burnett et al. [49] by estimating the premature death reduction attributable to the ambient reduction in PM_2.5_ concentration. This model has been conducted by calculating global mortality of ambient and household air pollution in 2010 based on the parameter determination derived from the Global Burden of Disease (GBD) project [37,49].

The mortality of PM_2.5_ concentration estimated in the IER model is determined by Equation (7),

(7) $$\begin{array}{ll} RR(C_{m}) & =1+\alpha [1-\exp(-\gamma ({C_{m}-C_{0})}^{\delta })]\;for\;C_{m}>C_{0},\\ & =1\;\;\;\;\;\;\;\;\;\;\;for\;C_{m}\leq C_{0}. \end{array}$$

In Equation (7), *RR* means the relative mortality attributed to PM_2.5_ concentration. The deaths attributed to PM_2.5_ concentration are derived from four leading diseases, that is, cerebrovascular disease (stroke, CEV), ischemic heart disease (IHD), chronic obstructive pulmonary disease (COPD), and lung cancer (LC) [37,49]. In which, $C_{m}$ denotes the annual mean PM_2.5_ concentration, ranging from 17.17 to 106.25 Mcg/m^3^ in this study; $C_{0}$ is the theoretical lower bound of risk exposure concentration [50], which is assumed to be 5.8 Mcg/m^3^ [37]. α, γ and δ are parameters related to the shape of the concentration–response curve of each disease [37,49,51]; the stochastic fitted values of three parameters were obtained from [52]. The *RR_q_* of disease *q* can be converted by the attributable fraction (AF) [51] in Equation (8).

(8) $$AF_{q}=(RR_{q}-1)/RR_{q}.$$

The excessive mortality (i.e., the $\Delta E_{mort}$) attributable to PM_2.5_ concentration $C_{m}$ is further estimated in Equation (9) based on the *AF**_q_*** obtained from Equation (8).

(9) $$\Delta E_{mort}{\left(C_{m}\right)}_{iq}=(AF_{q}\times BIR_{q}\times EP_{i})/EP_{i}=AF_{q}\times BIR_{q}.$$

*BIR* denotes the baseline incidence rate of a given health impact in Equation (9), which is cause-specific mortality rate per unit population for the whole age group. The baseline mortality (i.e., *BIR*), following Maji et al. [37], is defined for stroke, IHD, COPD, and LC among China’s cities, respectively. *EP* means the PM_2.5_ emission-exposed population. More details of the IER model are discussed in [37,49,51]. 

Using the estimation from the overall perspective as an example, based on the obtained excessive mortality $\Delta E_{mort}$, the potential mortality reduction of specific diseased *q* caused by PM_2.5_ concentration is achieved by

(10) $$PR_{mort}^{iq}=\Delta E_{mort}{\left(C_{m}\right)}_{iq}-\Delta E_{mort}(\sum_{j=1}^{n}\eta_{j}S_{j}/t{)}_{iq}.$$

Where $PR_{mort}^{iq}$ represents the potential reduction gap in public mortality between the actual excessive mortality of $\Delta E_{mort}{\left(C_{m}\right)}_{iq}$ and expected excessive mortality of $\Delta E_{mort}(\sum_{j=1}^{n}\eta_{j}S_{j}/t{)}_{iq}$. Here $\sum_{j=1}^{n}\eta_{j}S_{j}/t$ denotes the optimal excessive PM_2.5_ concentration estimated by DEA. A similar estimation can also be conducted from the environmental perspective.

**Figure 2:**
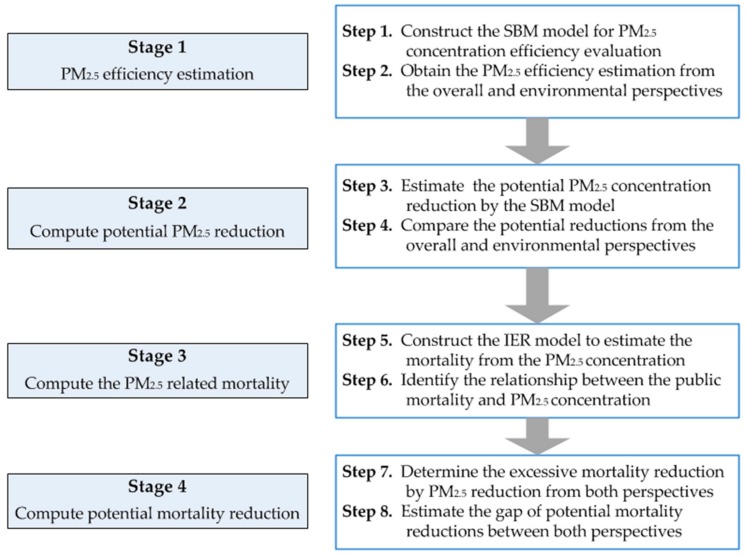
Flowchart of the proposed methodology.

A method framework has been systematically developed to illustrate the proposed estimation. The methodology can be introduced in the following stages: (1) estimating the PM_2.5_ concentration efficiency by SBM model from the overall and environmental perspectives; (2) determining the potential PM_2.5_ concentration reductions from both perspectives; (3) computing the total mortality rate of all the diseases caused by excessive PM_2.5_ concentration; and (4) estimating the potential public mortality reduction attributable to PM_2.5_ concentration from both perspectives. A detailed diagrammatic description of the proposed method framework is shown in Figure 2.

## 3. Results 

### 3.1. The Particulate Matter 2.5 _2.5_ Concentration Efficiency and Potential Mortality Reduction for Different Areas of China

Based on the obtained data, the PM_2.5_ concentration efficiency in models (2) and (4) can be obtained, respectively. An areal comparison of the mean urban PM_2.5_ concentration efficiency from the overall and environmental perspectives is provided as follows. 

**Figure 3:**
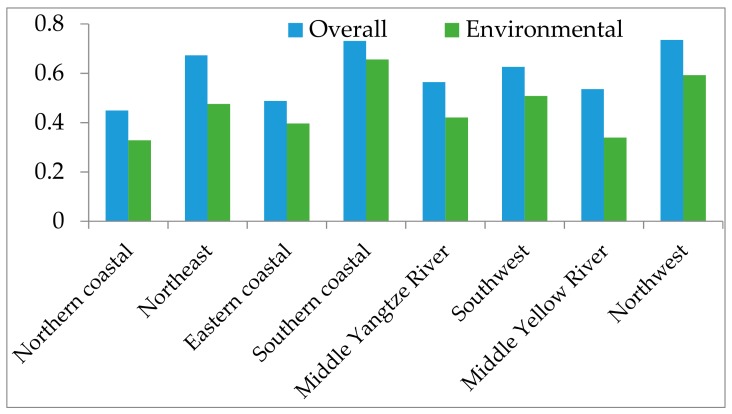
Mean urban PM_2.5_ efficiencies for different areas in China.

Figure 3 shows that all the mean PM_2.5_ concentration efficiencies from the overall perspective are higher than from the environmental perspective. The northwest area (i.e., 0.7351 and 0.5922, respectively) and southern coastal area (i.e., 0.7309 and 0.6560, respectively) have higher mean PM_2.5_ concentration efficiencies than other areas from both the overall and environmental perspectives, respectively. Conversely, the northern coastal area has the lowest mean PM_2.5_ concentration efficiency (i.e., 0.4490 and 0.3285, respectively) among all the areas from both perspectives. This indicates that the southern coastal area and northwest area have the best potential PM_2.5_ pollutant reductions in China, and vice versa for the north coastal area. This result could be partially explained by the corresponding mean urban PM_2.5_ concentration. The north coastal area has the highest mean urban PM_2.5_ concentration (i.e., 76.38 Mcg/m^3^) among all areas; the southern coastal area has the lowest urban PM_2.5_ concentration (i.e., 31.08 Mcg/m^3^).

**Figure 4:**
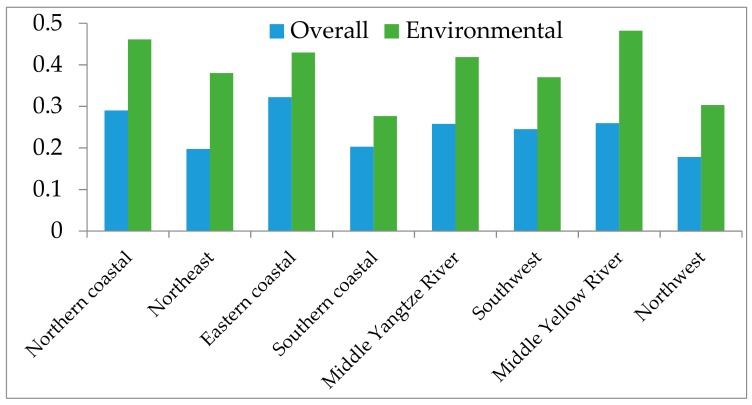
Potential urban public mortality reduction ratio caused by the PM_2.5_ concentration for areas.

To analyze the public mortality caused by excessive PM_2.5_ concentration, this paper applied Equation (10). The actual public mortality was derived from four leading diseases which occurred among the studied Chinese areas in 2015. The ratios of potential mortality reduction to the actual mortality from the overall and environmental perspectives are illustrated in Figure 4.

Comparing the areal potential mortality reduction ratios, Figure 4 reveals that the eastern coastal area had the largest ratio (i.e., 32.19%) among all the areas from the overall perspective, followed by the northern coastal area (i.e., 29.02%). Conversely, the northwest area had the least potential mortality reduction ratio (i.e., 17.81%) from the overall perspective. From the environmental perspective, the largest reduction ratio is observed in the middle Yellow River area (i.e., 48.22%), while the southern coastal area had the lowest reduction ratio (i.e., 27.67%). There are significant discrepancies in potential reduction amounts between the overall and environmental perspectives for all the areas, which further proves Proposition 1. Interestingly, a non-initial implication could be described. For example, it was discovered that the eastern coastal area (i.e., 0.4877) had higher PM_2.5_ concentration efficiency than the northern coastal area (i.e., 0.4490) from the overall perspective, while the potential mortality reduction ratio in the northern coastal area (i.e., 29.02%) was discovered to be less than that in the eastern coastal area (i.e., 32.19%) from the overall perspective. This was opposite to the general concept that the DMUs with larger potential reductions would have lower efficiency scores. To explain this non-intuitive result, the relationship between the PM_2.5_ concentration and public mortality has been further explored. 

**Figure 5:**
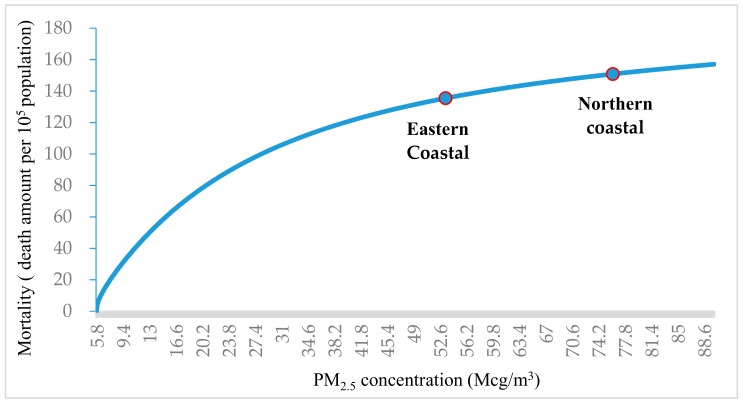
The relationship between PM_2.5_ concentration and public mortality.

Figure 5 demonstrates the relationship between PM_2.5_ concentration and public mortality. The horizontal axis represents the PM_2.5_ concentration (Mcg/m^3^). The vertical axis denotes the public mortality rate. The slope of the curve becomes gentler as the PM_2.5_ concentration increases. This indicates that if the specific area A is on the left of area B in the curve, the constant PM_2.5_ concentration reduction in area A results in greater potential public mortality than in area B. In this case, the position of the northern coastal area is to the right of the eastern coastal area in Figure 5. The actual mean PM_2.5_ concentration of the northern coastal area (i.e., 76.38 Mcg/m^3^) was larger than that of the eastern coastal area (i.e., 53.34 Mcg/m^3^). Thus, the constant PM_2.5_ concentration reduction in the eastern coastal area could induce more potential public mortality reduction than in the northern coastal area. This can help to explain the non-intuitive implication. A similar finding in the relationship between the PM_2.5_ concentration and public mortality has also been proposed by Li et al. [53].

### 3.2. The Particulate Matter 2.5_2.5_ Concentration Efficiency and Potential Mortality Reduction for China’s Cities

**Figure 6:**
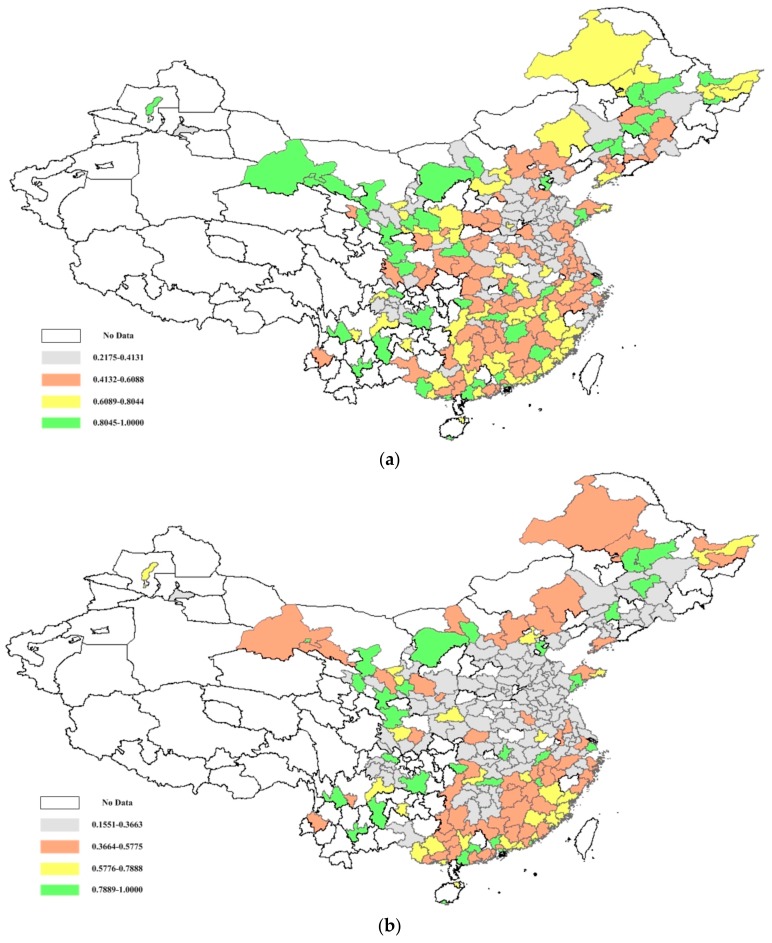
China’s urban PM_2.5_ efficiency from overall (**a**) and environmental (**b**) perspectives.

**Figure 7:**
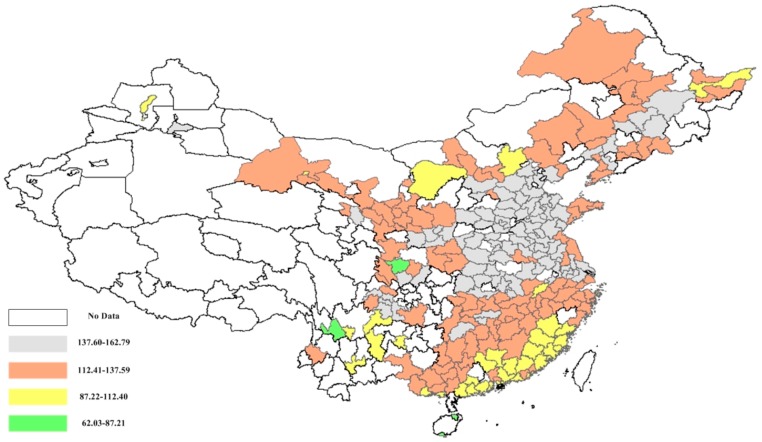
The actual mortality caused by excessive PM_2.5_ emission in urban China.

**Figure 8:**
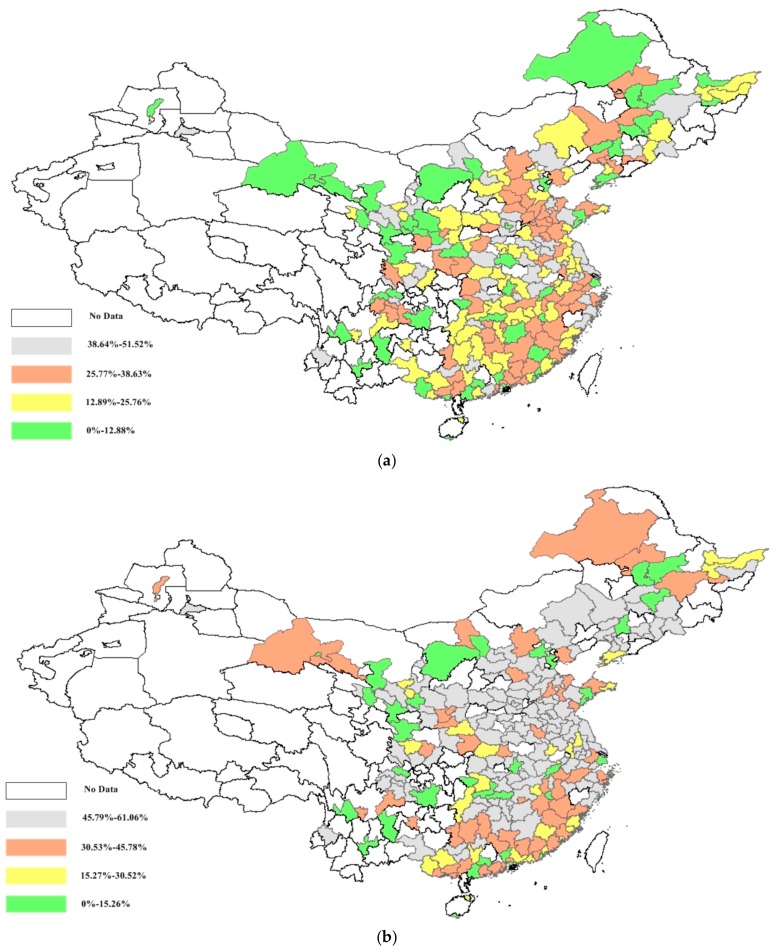
Potential mortality reductions from overall (**a**) and environmental (**b**) perspectives.

The urban illustrations of the PM_2.5_ concentration efficiency and potential public mortality reduction are further presented in Figure 6, Figure 7 and Figure 8. Figure 6 shows the PM_2.5_ concentration efficiency scores from the overall and environmental perspectives. Regarding the overall perspective, 32 cities are observed to have significant efficiency scores, which are distributed among China’s cities. The mean PM_2.5_ concentration efficiency score among China’s cities is 0.5899, which reveals that most of China’s cities have the potential to reduce the PM_2.5_ concentration. Twenty-six cities with high efficiency are presented from the environmental perspective.

Notably, there is a significant regional disparity in the distribution of urban PM_2.5_ concentration efficiencies. Figure 6 shows that the cities with lower efficiency scores (less than 0.4131) are mainly agglomerated in the northern coastal area and eastern coastal area, which accounts for 48.15% of the total cities within these areas. It is larger than the mean level in China (i.e., 25.93%). The cities with higher efficiency scores (larger than 0.8045) are mainly located in the northwest and northeast, accounting for 43.90% of the total cities, and are larger than the national mean (i.e., 18.52%). A similar distribution trend can also be seen from the environmental perspective. As observed from the urban illustration, the lower PM_2.5_ concentration efficiency agglomeration mainly exists in the cities of the northern coastal area and eastern coastal area, which shows a decreasing trend from the east to the west.

Furthermore, the actual public mortality caused by PM_2.5_ concentration was measured at the city level. Based on the estimation in Equations (7)–(10), the public mortality rates among China’s cities are depicted in Figure 7. Figure 7 shows the significant geographic agglomeration of the urban public mortality. The northern coastal area had the largest mortality (i.e., 144.08 people/10^5^ people) among all the areas, and the second highest was the middle Yellow River area (i.e., 139.30 people/10^5^ people). The cities with higher mortality were mainly agglomerated in the northern coastal area and middle Yellow River area. The higher urban mortality was explained by these regions being the main energy consumers in China, accounting for 29.11% of China’s total energy consumption. Furthermore, most cities with lower mortality (i.e., 60.98% of cities with the mortality lower than 112.41) were mainly located in the southern coastal area. No cites with mortality higher than 137.60 were observed in the southern coastal area. Similar to the findings of Chen et al. [54], the distribution of actual mortality was consistent with the PM_2.5_ concentration efficiency from the overall perspective.

Based on the obtained PM_2.5_ concentration efficiency and actual public mortality, the potential public mortality reductions among cities were analyzed from the overall and environmental perspectives. Regarding the overall perspective, 16.87% of cities could reduce more than 40% of the potential mortality; while from the environmental perspective, 64.20% of cities could reduce the potential mortality by more than 40%. Moreover, 32 cities were discovered with no potential mortality reductions from the overall perspective. Regarding the environmental perspective, 26 cities (included in the 32 from the overall perspective) were to have zero potential reductions. These cities could be inferred to have less public mortality impact from PM_2.5_ emissions due to their lower pollution, which were dispersedly distributed among China’s areas. It was discovered that all the cities had higher potential mortality reductions from the environmental perspective than that from the overall perspective.

Significant geographic disparity is shown in the distribution of potential mortality reductions in Figure 8. Concerning the overall perspective, it was observed that most cities with larger potential mortality reduction (larger than 38.64%) are agglomerated in the eastern coastal area and middle Yellow River area, accounting for 27.59% of the cities in both areas, which is larger than the national level (i.e., 19.75%). The northwest and northeast areas have the most cities with lower potential mortality reduction (less than 12.88%) among all the areas, which accounts for 48.78% of all the cities, larger than the national level (i.e., 21.40%). Notably, the cities in the Sichuan province show significantly worse actual mortality and potential mortality reduction (i.e., 130.42 and 0.2870) than the mean areal level (i.e., 121.65 and 0.2218) in west China (i.e., northwest and southwest areas). Figure 8 shows that more potential public mortality reduction could be achieved in cities along the eastern coastal area and middle Yellow River area in China. A decreasing distribution trend of urban potential mortality reduction, similar to that of the PM_2.5_ concentration efficiency, is also witnessed from the east to west China from both perspectives. 

**Figure 9:**
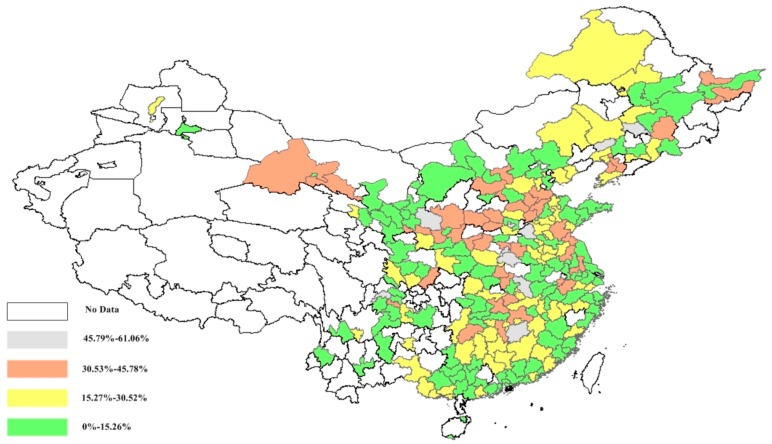
Potential mortality reduction gaps between overall and environmental perspectives.

Note that the gaps between the potential mortality reduction from the overall and environmental perspectives are illustrated in Figure 9. The gaps here reflect the potential mortality reduction by addressing the environmental target at the cost of the economic output. Thirty-three cities were discovered to have no potential reduction across China from either perspective. The cities of Fuxin, Siping, Meishan, Qingyang, Hebi, Zhumadian, Jian, and Luohe had reduction gaps accounting for over 40% of the total reduction. Approximately 57% of cities with higher reduction gaps (i.e., over the gap of 30.53%) were distributed in central China (i.e., middle Yangtze River area and middle Yellow River area). This result could explain by the existence of pollutant-intensive economic development in these cities. More PM_2.5_ emission allowances could accelerate the economic growth of these regional production forms. Therefore, to achieve improvement in public health, the cities with larger potential reduction gaps should attempt to reduce the PM_2.5_ emission at the cost of their economic growth. 

Interestingly, these six cities, Beijing, Ziyang, Wuwei, Urumqi, Zhongshan, Dongguan, and Tongling were observed to have no potential gaps but some potential reductions. This reflects that these cities would have constant potential mortality reductions regardless of their economic output. This reveals that the reduction gaps were caused by technical defects in reducing the PM_2.5_ concentration rather than production resource adjustment for these cities. The appropriate environmental efforts were already launched by adopting the existing production resource. The main focus of these cities for reducing the public mortality is to update the current technology of PM_2.5_ concentration reduction.

## 4. Discussion

According to the obtained PM_2.5_ concentration efficiency and corresponding potential public mortality reductions, some implications have been found. In the PM_2.5_ concentration efficiency estimation, the northern coastal area, eastern coastal area, and the middle Yellow River area have the lowest PM_2.5_ concentration efficiency and the largest potential reduction in public mortality in China. The PM_2.5_ concentration reduction should be addressed in these areas. Note that the southern coastal area and northwest area are observed to have the highest PM_2.5_ concentration efficiency. The southern coastal area is also the economically developed area (accounting for 41.39% of urban GDP output for all the observed cities) with better PM_2.5_ pollutant conditions in China. Thus, the environmental production experience in the southern coastal area could be useful for all of China. 

Interestingly, the eastern coastal area was discovered to have higher PM_2.5_ concentration efficiency and larger public mortality reduction than the northern coastal area. This is a non-intuitive implication, which could be explained by identifying the relationship between the PM_2.5_ concentration and public mortality. This indicates that the regional public mortality is consistent with the PM_2.5_ concentration, but some inconsistency also exists.

Concerning the urban vision, the cities having worse PM_2.5_ concentration efficiencies are located mainly in the northern coastal and eastern coastal areas, while cities with higher public mortality reduction are mainly agglomerated in the eastern coastal and middle Yellow River areas. A significant trend of increasing PM_2.5_ efficiency and decreasing potential mortality reduction can be witnessed from the east to the west among China’s cities. Notably, in west China, the cities in Sichuan province show significantly worse potential mortality reduction. Based on Cheng et al. [55], high heavy metal factors were observed in airborne dust in Sichuan (i.e., Chengdu), which could be adsorbed easily by PM_2.5_ which is harmful to public health. This could be caused by the large quantity of automobiles (i.e., 7.6713 million units, largest in west China) and pollutant-intensive industrial infrastructure (i.e., the heavy industrial production accounts for 66.67% of the total industrial production) in Sichuan in 2015. To improve public health, the PM_2.5_ mortality reduction should be addressed, and the government should make efforts to reduce the heavy metal rate of PM_2.5_ emission (e.g., discarding the pollutant-intensive industrial sectors or advocating for waste gas purification), especially for Sichuan. Importantly, comparing results in areal and urban vision, a significant difference could be detected. An example is urban Zhuhai and Jiangmen within the Guangdong province, which show inefficient scores in the PM_2.5_ efficiencies from the overall perspective (i.e., 0.5908 and 0.5002, respectively). The Guangdong province would have low PM_2.5_ concentration efficiency in this case, while Shenzhen and Guangzhou are in Guangdong and show high PM_2.5_ concentration efficiency scores. This indicates that some cities in inefficient provinces or areas can be efficient at the city level. Thus, urban illustration could provide a clearer vision in determining the PM_2.5_ concentration inefficiency and corresponding public health improvement than provincial or areal illustration. Additionally, detailed improvement solutions can be proposed depending on the local urban condition, which could help to reduce the local PM_2.5_ concentration and improve relative public mortality. 

Based on the comparison between the overall and environmental perspectives, the potential reduction gaps in public mortality have been explored. These gaps could reflect the potential improvement in public mortality reduction by launching efforts to reduce the PM_2.5_ concentration in ignorance of economic growth. Based on the comparison, it is discovered that the PM_2.5_ concentration should be reduced at the cost of economic output in central China. This can be explained by the pollutant-intensive industrial structure in central China. The PM_2.5_ intensity (i.e., the ratio of PM_2.5_ concentration to the GDP output) in central China (i.e.,0.0538 Mcg/m^3^ per billion yuan) is larger than that of the whole country (i.e.,0.0296 Mcg/m^3^ per billion yuan ). Central China has the feature of economic production dependence on PM_2.5_ emission. Notably, some cities with poor PM_2.5_ efficiency have been proven to have no gaps in public mortality reductions between the two perspectives. The updating of clean-production technology for these cities should be advocated for, rather than resource consumption adjustment.

However, the PM_2.5_ concentration data is collected from outdoor monitoring sites. Considering the available data, the corresponding mortality is correlated with the outdoor PM_2.5_ pollution. However, the indoor PM_2.5_ concentration, which is generated from indoor living activities (e.g., cooking and smoking) or is derived from the outdoor pollution [56], is also a factor. The indoor PM_2.5_ concentration could also result in serious public mortality due to the potential for accumulation of microorganisms on indoor PM_2.5_ [56]. Detailed investigation of mortality reduction might provide a more interesting issue in the future. Furthermore, this study is based on the available database of China’s 243 cities in 2015. A study with a longer time-frame and more urban observation could extend this study, giving more insights into China’s urban PM_2.5_ efficiency and the corresponding public health.

## 5. Conclusions

This paper aimed to analyze the PM_2.5_ efficiency and the corresponding potential public mortality reduction in China. A method based on the SBM-DEA measure and IER model was proposed for this purpose. The SBM-DEA model was adopted to estimate the PM_2.5_ concentration efficiency from the overall and environmental perspectives. Identifying the relationship between the PM_2.5_ concentration and the public mortality in the IER model enables the potential public mortality results from the excessive PM_2.5_ concentration to be derived. The proposed method was applied to the empirical case of China’s cities from 2015.

According to the empirical study, several conclusions and implications were obtained. (1) From both areal and urban perspectives, regional diversity in the results of PM_2.5_ concentration efficiency and potential mortality reduction were observed. Emission reduction and public health improvement should be advocated for in all of China’s cities based on the local urban condition. (2) Some inconsistency between the distribution of PM_2.5_ concentration efficiency and public mortality reduction was discovered by identifying the relationship between PM_2.5_ concentration and public mortality. (3) Production adjustment with environmental consideration should be advocated for in most of China’s cities to further improve the conditions of PM_2.5_ pollution and public health. However, technological updating is more urgent than production adjustment for some cities which have no gaps in the potential mortality reduction between overall and environmental perspectives. 

Some policy suggestions can be made towards potential mortality reductions in urban China. First, the government should launch more policies and regulations regarding the reduction of PM_2.5_ concentration and potential mortality for China’s cities, especially for cities in the eastern, northern, middle Yellow River areas, and Sichuan province in west China. The more environmentally-friendly production experience in the southern coastal area, such as the industrial structure and the economic development model, could be advocated for in other areas in China. Notably, attempts to remove the microorganism and heavy metals mixed (e.g., the filtration technology adoption) in the PM_2.5_ emission should be addressed as an important environmental solution for public health. Second, environmental consideration in economic production should be addressed in urban central China; for example, the energy-intensive industrial sectors in central China should be gradually dismantled. Third, adopting advanced energy conversion technologies or updating clean production technologies should be advocated for to improve the public health condition in cities without reduction gaps between perspectives (i.e., Beijing, Ziyang, Wuwei, Urumqi, Zhongshan, Dongguan, and Tongling). Importantly, an urban vision for local total-factor production should be accepted to set specific solutions for the PM_2.5_ concentration reduction and public health improvement by the government. 

## Figures and Tables

**Table 1:** Descriptive statistics.

Factor	Unit	Max	Min	Mean	Std.Dev
Capital	10^8^ CNY	1729.0757	2.4504	80.290327	169.0966
Employee	10^4^ people	35,271.782	396.6172	5054.277	5102.978
Energy	10^4^ TCE ^1^	3155.1309	2.496094	177.63696	333.3128
GDP	10^8^ CNY	24,838.37	70.2111	1560.4235	3122.897
PM_2.5_	Mcg/m^3^	106.25	15.75	52.586283	17.72751

^1^ TCE ton coal equivalent.

## Supplementary Materials

The following are available online at www.mdpi.com/1660-4601/15/3/529/s1, Table S1: Employee, capital stock, energy consumption, GDP, PM_2.5_ concentration and annual mean population of 243 prefecture level and above cities in China in 2015.
